# Current News and Opinion

**Published:** 1887-08

**Authors:** 


### Current News and Opinion.

### OBITUARY.

### J. R. WALKER, D. D. S.,

Died at his residence, Bay St. Louis, Miss., June 22, 1887, in the fifty-seventh year of his age. Dr. Walker was born in the State of New York, but obtained his dental training in Illinois and Michigan, finishing as a pupil of Dr. Foster of Jackson. In 1858 he went to New Orleans, where, if we except the time spent in the Confederate service during the war, he has ever since been in practice, and has held a leading position. He has always been prominent in dental societies, and was a frequent attendant upon their meetings. It was not alone in dental science that he was interested. He was a valued member of the American Association for the Advancement of Science, and read several interesting and excellent papers before that organization. Dr. Walker had strong and positive views upon most professional subjects of the day, and urged them with great vigor and ability. He was a sententious and logical reasoner, and his published essays show him as a clear and lucid writer. That he possessed the respect of his professional associates is proved by the many positions of honor to which he was advanced. For some time he had been failing in health, as those who met him at society meetings could plainly perceive. His strength continued to diminish, until at the last he passed away as the weary child sinks to quiet sleep.

### THE INTERNATIONAL MEDICAL CONGRESS.

Treasury Department,<br>

Office of the Secretary,<br>

Washington, D. C., June, 1887.

*Prof. John B. Hamilton, M. D., Secretary-General Ninth International Medical Congress, Washington, D. C.:*

Sir—In reply to the inquiries contained in the letter of F. H. Rehwinkel, M. D., referred by you to this department, I have to state that the professional books, implements, instruments, etc., of persons arriving in the United States, are exempt from duty under the provisions of the tariff law, which also provides for the free admission of "models of inventions and other improvements in the arts, such as cannot be fitted for use;" and that parties arriving from foreign countries for the purpose of attending the Ninth International Medical Convention, to meet at Washington, September 5, 1887, and who bring with them their own "surgical or dental instruments, scientific and mechanical appliances, models and materials to be used for clinical demonstration under the direction of the said Congress," will be entitled to have the same passed free of duty, on the usual oath that they are for their personal use and are not intended for sale.

As it would appear from the act of Congress relative to said convention, that the said Ninth International Medical Congress is a society established for philosophical and scientific purposes, books, maps and charts (not more than two copies in any one invoice), philosophical and scientific apparatus, instruments and preparations, casts, paintings, drawings and etchings, specially imported in good faith for the use of said Congress and not intended for sale, would also seem to be entitled to free entry under the provisions of the tariff act now in force, a copy of which is herewith enclosed. (See paragraphs 660, 759 and 815.)

Copies of this letter will be transmitted to the collectors at the principal ports for their information and guidance.

Respectfully yours,

C. S. Fairchild,
Secretary of the Treasury.

---

### ADMISSION OF DENTISTS TO THE CONGRESS.

*Editor Independent Practitioner:*

Dr. Hunt, of Washington, and the writer, called on Dr. J. B. Hamilton, Secretary-General of the Ninth International Medical Congress, at his office, and there met two or three members of the Registration Committee of the Congress.

We were authorized to say that the Registration Committee considered the question of the admission of dentists to membership in the Congress clearly and definitely settled by the resolution adopted by the American Medical Association, and that, in accordance with the resolution, dental graduates can register and take out tickets of admission the same as medical graduates, and that a form of registration has been prepared, in which dentists will register as special practitioners.

Will you please insert this in the August number of the Independent Practitioner, for general information, and also say that the meetings of Section XVII will be held in the "Church of Our Father," corner of 13th and L streets, and the clinics and exhibits in the Franklin School Building, corner of 13th and K streets.

C. F. W. Bodecker, D. D. S., M. D. S.

### REGISTRATION BLANK.

"This Congress will consist of such members of the regular medical profession as shall have registered and taken out their tickets of admission, and of such other scientific men as the Executive Committee of the Congress shall deem it desirable to admit."

Those about to register will please read and give attention to the following directions to secure accuracy in the list of members of the Congress, and to prevent mistakes therein it is especially requested that all proper names of persons and places shall be written distinctly and in full, and without abbreviations in any case. (*For example:* Instead of J. W. Taylor, Phila., Pa., it should be written John Warren Taylor, Philadelphia, Pennsylvania, etc., etc.

By order of

The Committee of Arrangements.

*Register No*...............

FORM TO BE FILLED UP BY APPLICANT FOR MEMBERSHIP IN THE CONGRESS.

DATA REQUIRED :

Name in full..................................................................

Post-Office Address............................................................

If in city, give also street and number.......................................

If in the country, give also name of county ..................................

State...............................

Province...............................

Country...............................

If a Delegate, state from what country or society..............................

Practice general or special ...................................................

If special, name the branch....................................................

---

*Editor Independent Practitioner :*

Will you please give notice that, as I am unable to continue the work of the Secretaryship of the Dental and Oral Section of the International Medical Congress, Dr. A. M. Dudley, of Salem, Mass., has kindly consented to act.

All communications should henceforward be addressed to him.

E. A. BOGUE.

---

### NOTCHES IN THE UPPER CENTRAL INCISOR TEETH WHICH RESEMBLE THOSE OF SYPHILIS.

There is a state of notching of the upper incisor teeth, which affects the two central ones of the permanent set, and produces a condition very deceptively like that of syphilis. The notches are central, and very conspicuous. A chief point of difference from the syphilitic tooth is that the tooth is usually wide instead of narrow at its free edge. Syphilitic teeth almost always show narrowing, like a screw-driver, as well as notching.

Another point of difference is that the teeth, when looked at carefully, are seen to be craggy and very hard, not worn as the syphilitic tooth. In a very marked example of the pseudo-syphilitic notching, the father of the patient told me that the condition was hereditary, and the youth's mother had teeth of the same kind. In this instance there was no history of fits in infancy, or of the use of mercury or teething powders. Nor, indeed, were the conditions those of stomatitis, or mercurial teeth. The defects occurred in pairs of teeth, and did not damage the whole row. Nor were the first permanent molars—the test teeth of the mercurial set—involved. I have in several other examples of craggy teeth been assured that the peculiarity was in the family. I feel certain, therefore, that we must admit inheritance as an occasional explanation of peculiarities in the form of the teeth. I was once shown, in one of the Paris hospitals, a pair of teeth such as those which I have above described, and great surprise was expressed that I could not admit that they were characteristically syphilitic.—*Jonathan Hutchinson in British Medical Journal.*

### PYORRHŒA ALVEOLARIS.

Mr. Newton Pedley, F. R. C. S., read a paper at the Odontological Society upon the above subject. Pyorrhœa alveolaris is characterized by conditions as follows: The mucous membrane, especially that adjacent to the teeth, is deeply congested, tumid and thickened, and detached from the necks of the teeth and from the roots. A thick, fetid discharge may often be pressed up from between the teeth and the mucous membrane, which gives the breath a very repulsive odor. Later the alveoli become absorbed and at times more or less denuded, while the fangs of the teeth become coated with a hard, thick, green-brown tartar. Ultimately, the disease progressing, the teeth, one after another, drop out. The pathological changes which take place are hypertrophy of the muco periosteal fold around the teeth, accompanied by dilatation of capillary loops, enlargement of the papillæ, and rapid proliferation of epithelial cells. Later the gum becomes firm and contracted and displays increase of fibrous tissue. The changes which go on in the socket have not yet been satisfactorily worked out, but the examination of the jaws of some carnivora which were apparently affected with pyorrhœa alveolaris would lead to the supposition that there is osteitis of the alveolar process spreading toward the apex of the socket. There are many differences of opinion as to the causes, some maintaining that it is of parasitic origin, and due to a specific bacillus, but there is no good proof of this; others that it is catarrhal, and an extension of inflammation to the mucous membrane; others that it is due to small deposits of hard tartar under the edge of the gum, but this is plainly not the case, for the disease may be far in advance of the deposit, and in some instances there is not any to be found. It is probably due to some constitutional condition, and the fact that it is often symmetrical and frequently hereditary gives support to this view. It occurs in the mouths of patients whose health has been undermined by debilitating influences and injudicious habits of living; it is a common sequel of malarial fever in America; young persons recovering from eruptive fevers are sometimes victims of pyorrhœa alveolaris, and frequent pregnancies are a fruitful source of the disorder. Attention has lately been drawn to shedding of the teeth in tabes dorsalis, but it does not by any means seem to be a constant symptom. Mr. Bland Sutton has found that premature loss of the teeth is a very common affection in cases of rheumoid arthritis in animals, and has also met with it in mollities ossium and other wasting diseases. Magitot, who views the alveolar-dental periosteum as a ligament, and not of the same nature as osseous periosteum, calls the disease symptomatic alveolo-arthritis, and mentions especially as causes chronic Bright's disease and glycosuria, in which latter, he says, the phenomenon is absolutely constant.—*The Lancet.*

---

### HOW TO CHOOSE A DOCTOR.

To be a doctor, one must first be a man, and a mean man cannot be a good doctor any more than he can be a good minister or a good husband, and a really honest, large and loving man cannot make a poor doctor, no matter what his pet party may be. To have good sense as a doctor, one must have good sense as a man. If your doctor is a nincompoop about other things you may be sure that he is a ninny as to medicine and surgery. If the doctor's office is untidy and vile to smell of, you may be quite certain that he will come short of giving good counsel as to health and tidiness of body. If he be clumsy in hitching his horse you may be sure that he is not handy at surgery midwifery. If he be a great, coarse, blundering fellow, careless of dress, a two fisted, farmer-looking man, you may be sure he will lack perception of those finer symptoms by which a good doctor is guided. If he slanders brother physicians who profess a different party, you may be sure that he is himself a quack. Good earnest doctors are too busy to find time to slander their brethren or their rivals. It is all the same with lawyers, ministers and teachers. The truly good and truly great do not detract from the reputation of others, and they are generous and magnanimous even to rivals. If your doctor falters you, and humors your lusts and appetites, and helps you out of a bad scrape secretly, without reproof, as if you had done no wrong, distrust him. If you can hire him to do or say what he would not do without the hire, beware of him. Good doctors cannot be bought. Your doctor ought not to be a single man. He ought to have a wife and children, and if you see that his wife respects him, and his children obey him, that is a very good sign that he may be trusted. If your doctor tells you how to keep well, that is a good sign. You come to him with the toothache ; he gives you creosote and clove oil for the tooth, and at the same time suggest that you do not wash enough to keep well—that is a good sign. If the children like him, that is a good sign. If you find him reading in his office, that is a good sign, especially if he be a settled middle-aged man. If you hear him say : "I once thought so and so, but I was wrong," that is a good sign. If the doctor is neat and handy in rolling pills and folding powders, that is to his credit as a surgeon. If he understands how to bud roses, graft fruit trees, mix strawberry pollen for improved berries, cure chicken pip, and tinker a trunk lock, or put a clock in order, all these are so much to his credit. If, further, you love to meet him, the sight of him quickens you, and you are glad to hear him chat, and you know him to be a lovable, sympathetic man—he's the man for your doctor, your confidential friend; find him, trust him—*Beecher.*

---

### MISSOURI STATE DENTAL ASSOCIATION.

The twenty-third annual meeting of the Missouri State Dental Association was held at Kansas City, June 21 to 24. The following officers were elected for 1888:

*President*—Wm. N. Morrison, St. Louis.
*First Vice-President*—T. M. Nicholson, Fayette.
*Second Vice-President*—J. T. McWilliams, Mexico.
*Recording Secretary*—John G. Harper, St. Louis.
*Corresponding Secretary*—Wm. Conrad, St. Louis.
*Treasurer*—James A. Price, Weston.

The next meeting will be held at Perle Springs, Warrensburg, Mo., the first Tuesday after July 4, 1888. WILLIAM CONRAD, Cor. Sec'y.

### SOUTHERN DENTAL ASSOCIATION.

The meeting of the Southern Dental Association, at the Hygeia Hotel, Old Point Comfort, Va., August 30th, promises to be a grand success, if not the largest meeting of the dental profession on the continent. The Central Traffic Association, as well as the Southern Passenger's Association, will give reduced rates to those who wish to attend. Those purchasing tickets from Central Traffic Association agents will get certificates from railroad agent when procuring tickets, which will enable the holder to return from "Old Point" for one-third fare. Those who wish to take advantage of the reduction offered by the Southern Passenger's Association will address Dr. J. Y. Crawford, Corresponding Secretary, Nashville, for certificates, which will enable the holder to make the trip by paying one and one-third fare. Tickets good twenty-four hours after adjournment of meeting. The Virginians and our grand old President, Dr. W. W. H. Thaxton, are exerting themselves to make the meeting a grand one. A cordial invitation is extended to all, east, west, north and south.

J. Y. Crawford, Cor. Sec.

---

### AMERICAN SOCIETY OF MICROSCOPISTS.

The Tenth Annual Meeting of the Society will be held in Pittsburgh, Penn., beginning August 30th, and lasting four days. The time is set for the week preceding the meeting of the International Medical Congress at Washington, and will therefore be convenient in both time and place for those who desire to attend both conventions. It is hoped that we shall have the pleasure of welcoming at this meeting distinguished men of science from abroad as well as from our own country, who may be on their way to the Medical Congress.

The arrangements made by the local committee are such as to secure a most agreeable social reunion and interesting general and special sessions. Hotel headquarters will be at the Monongahela House, and the sessions will be held in the chapel of the First Presbyterian Church.

D. S. Kellicott, Secretary.

William A. Rogers, President.

---

### ANOTHER CONUNDRUM.

Why is it that in about ninety-five per cent. of cases, a cut of the upper jaw shows more depression upon the left side in the region of the cuspid tooth than upon the right, requiring more filling up to restore contour, and longer teeth, in order to have the ends of the teeth level?

I have observed this for many years, and have asked many dentists the reason why, but have received no reply.

The only reason I can see for it is this: The most of people are *right-handed*, biting off food at that corner of the mouth; if the teeth are in good condition, *chewing* more upon that side than the other, and as a result the bone is developed more upon the right side.

Is this the cause? If not, will some one "rise and explain?"

L. P. Haskell.

### GERMAN DENTAL TITLES.

Before the consolidation of the German Empire every man who entered upon business, whether professional or secular, was required to take out the proper license. When the Empire was formed, all except those who practiced a profession were freed from this obligation. Dentists were not considered as practicing a profession, but when it was seen that all classes of people were entering upon dental practice, the mistake was recognized and a law was passed requiring them to be licensed. A fee bill was established by law, but it was made so ridiculously low that it is quite impossible to practice by it, even the veriest quacks demanding more. For instance, the fee bill allowed by law only gave to the dentist from thirty-seven to fifty cents for filling a tooth, and the same for regulating each tooth. The dentists' position in Germany has, therefore, always been an exceptional one.

The law not only determines fees, but it establishes grades of standing and reputation, Thus the Hof-Zahnarzt, or Zahnarzt-Doctor, may be said to be equivalent to the American M. D., D. D. S., the Zahnarzt to the D. D. S., while the Zahnkuenstler has no degree and is permitted to do only mechanical work. The title "Professor" is conferred by government, and the "Professor Doctor" is the highest distinction in the profession.

### A RIGHT UPPER CANINE TOOTH IN THE LEFT ORBIT.

Dr. John Ward Cousins reports in the *British Medical Journal*, April 23, 1887, a very interesting case of a child two years old, from whom he removed a tumor of the left orbit, which proved to be a supernumerary and misplaced right upper canine tooth. The crown of the tooth was enclosed in a sac, and the fang was attached to the orbital plate by fibro-cartilage. On examination the teeth in the mouth were found in a normal position, complete in number, and well formed. The jaws were also large and fully developed for a child two years old.

Cases of misplaced teeth, in strange situations, have often been recorded. These irregularities, however, are not associated with any special shape of jaw, or deficiency of size in the dental arch. The teeth are generally found to occupy an inverted position in the bone to which they are attached. They have been erupted in the hard palate and in the nares, but Dr. Cousins has been unable to find an instance on record of a tooth appearing in the orbit. The occurrence of a *right* upper canine in the *left* orbit is certainly singular, and this crossed displacement must have taken place at a very early stage of embryonic life.

### THE DIGESTIBILITY OF VARIOUS KINDS OF FOODS ACCORDING TO VANDERBECK.

*Meats.*—Easy to digest: mutton, venison, hare, sweetbread, chicken, turkey, partridge, pheasant, grouse, beef. Hard to digest: pork, veal, goose, liver, heart, brain, lamb, duck, salt meat, sausage. *Fish.*—Easy: turbot, haddock, flounder, sole, oysters, trout, pike. Hard: mackerel, eels, salmon, herring, salt fish, lobster, crabs, mussels, cod. *Vegetables.*—Easy: asparagus, French beans, cauliflower, beets, potatoes, lettuce. Hard: artichoke, celery, spinach, boiled cabbage. *Fruits, etc.*—Easy: baked apples, oranges, grapes, strawberries, peaches, cocoa, coffee, black tea, claret. Hard: apples, currants raspberries, apricots, pears, plums, cherries, pineapples, chocolate, pickles, beer.—*Journal of Reconstructives.*

---

Some curious evidences of the diet of our prehistoric ancestors of the "stone age" were recently brought before the Odontological Society of Great Britain, by Mr. Charters White. Mr. White was struck with the thought that, as particles of food become imprisoned in the dental tartar, sealed up in a calcareous cement, and can be made to reveal themselves on solution of this material, it would be an interesting revelation if the tartar found on these teeth of the stone age could be made to give up its secrets in a similar manner. He accordingly decalcified some with dilute hydrochloric acid and examined the sediment It consisted of masses, composed of epithelial scales mixed with the contents of starch cells. Besides these, Mr. White was able to identify portions of husks of corn, hairs from the outside of the husks, spiral vessels from vegetables, husks of starch, the point of a fish's tooth, a conglomeration of oval cells, probably of fruit, barblets of feathers, portions of wool and some fragments of cartilage, together with some other organic remains which he failed to recognize. The fact that vegetable tissue should be found in such a state as to be easily recognizable after the lapse of probably not less than three thousand years, is certainly remarkable.

---

A paternal government possesses some important advantages for the governed. It is not obliged to defer to every miserable demagogue who may be able to control a few votes, and it can afford to legislate against many petty and great swindles. There are in Germany, for instance, official chemists and examiners for foods, and every quack medicine is critically analyzed and its ingredients and character pitilessly exposed. A large proportion of these come from free America, where there is perfect liberty to commit fraud in these matters. Here is one exposition:

"Under the name '*Warner's Safe Cure*,' a brown liquid contained in flat bottles, holding about 500 grammes, is recommended as a remedy against kidney diseases, and sold at four marks per bottle. The official analysis, and the statement of a resident apothecary who sells the preparation, have shown that the article consists principally of American wintergreen, and its highest value is not over two marks. This notice is published for the information of the people."

Baron von Richtoven,
Berlin, March 30, 1887. President of Police.

---

Canadian dentists have not been frequent attendants upon the meetings of the American Dental Association. Even when we have met at Niagara or Detroit, upon the boundary line, but few have attended the sessions, while none have joined the Association, which has members from Long Island to San Francisco, and from Maine to Texas, but not one from Canada. Yet there is no reason why our Canadian brethren should not reap the advantages to be gained, as well as Americans. There should be a large delegation at the coming meeting at Niagara.

SCIENTIFIC MEN have been perplexed for many years over the phenomenon of a certain well at Yakutsk, Siberia. A Russian merchant in 1829 began to dig the well, but he gave up the task three years later, when he had dug down thirty feet and was still in solidly frozen soil. Then the Russian Academy of Sciences dug away at the well for months, but stopped when it had reached a depth of 382 feet, when the ground was still frozen as hard as a rock. In 1844 the academy had the temperature of the excavation carefully taken at various depths, and from these data it was estimated that the ground was frozen to a depth of 512 feet. Although the pole of the greatest cold is in this province of Yakutsk, not even the terrible severity of the Siberian winter could freeze the ground to a depth of 600 feet. Geologists have decided that the frozen valley of the lower Lena is a formation of the glacial period, They believe, in short, that it froze solidly then, and has never since had a chance to thaw out.—*National Druggist.*

---

THERE ARE AT PRESENT 1,435 people engaged in dentistry in the German Empire. Of this number, 505 are "approved" in Germany, 33 of whom are American graduates. Of the 94 practitioners who are graduates of American colleges, only 17 are females; 21 are "approved" in other countries, but not in America. There are 815 persons practicing mechanical dentistry without any other occupation. In the province of Hesse-Nassau there are 83 practitioners of dentistry, of whom 29 are "approved" in Germany, 4 being American graduates, and 14 from American schools exclusively, 3 of whom are females. Two of these are "approved" in other countries (not America), and 38 mechanical dentists without other occupation. In 77 cities with over 10,000 inhabitants there is not a dentist, surgical or mechanical, and in 86 cities there are no surgeons, though there are in these places practitioners of mechanical dentistry.

—*Jour. f. Zahnheilkunde.*

---

PROF. VALENTA recommends a simple procedure for keeping the hands soft. After washing and drying thoroughly, they are well anointed with cold cream; a small quantity of *spiritus saponatus* is poured in the hollow of one hand and this rubbed vigorously until a lather is produced; the fatty lather is merely rubbed off.

We have found no better preparation for the hands than the following, a little being taken in the hollow of one hand at night and thoroughly rubbed in. It should not be washed off till morning:—

℞

| | |
|---|---|
| Pow'd Borax, | gr. xl. |
| Glycerine, | |
| Rose Water, aa. | ℥ ii. |

---

SIR JAMES PAGET has been carefully studying the question "What becomes of the doctors," and reports that of 1,000 medical students whose careers were carefully followed, 23 attained eminence or gained leading practices; 66 had considerable success, gaining more than ordinary influence in their immediate vicinity; 507 were fairly successful, being able to live by their profession alone;

124 had an exceedingly limited practice, and 56 failed entirely; 96 (or nearly one-tenth) abandoned the profession entireiy; 87 died after entering practice, of whom 5 suicided, and 41 died while at college, most of them of phthisis, though two of them committed suicide. This compilation was made in England; in America the proportion that leave the profession to engage in other pursuits would be found to be much greater.

---

THE MONTREAL MEETING of the Connecticut Valley Dental Society was a memorable one on many accounts. Such hospitality and generous entertainment have seldom been tendered American dentists, and the visitors carried away the firm conviction that Montreal is the very home of good-fellowship. Indeed, it seemed as if there was no limit to the courtesies extended, and that Drs. Lovejoy, Bazin, Beers, Trestler, and—well, all the good fellows of Canada—had just determined to see what they could do when they really set themselves about the work. A great deal of commiseration was expressed for the few members of the Society who did not attend the meeting. In the next number of this journal will appear a report of the papers and discussions.

---

GERMAN DENTISTS are throwing every possible obstacle in the way of American dental college graduates, particularly those Germans who come here to obtain diplomas. They are not allowed to call themselves "Zahnarzt," nor to use the titles "Doctor" and "American Dentist" in combination. They may use either separately, but cannot use their German equivalents. It will not be long, probably, before American dentists will be forbidden to practice. For this result and for the degradation of the American degree, we have to thank certain American dental colleges which have conferred degrees almost indiscriminately upon foreigners, many of whom were entirely unacquainted with the English language.

---

BILLROTH writes the following concerning antiseptics:

1. Iodoform is the safest and most effective of all manageable antiseptics.
2. Moss, wood, turf, mould and oakum are useful when there are discharges from the wound.
3. Corrosive sublimate in dilute solution is practically inert as an antiseptic to wounds, and renders the patient and surgeon alike liable to mercurial poisoning.
4. Carbolic acid, which is known to be dangerous in strong solutions, in weak ones is as good for wound irrigations as clean water, but probably is no better.—*Canada Lancet.*

---

THE WESTERN MEDICAL REPORTER says that there are 131 medical journals published in the United States, exclusive of 52 devoted to dentistry, hygiene and pharmacy. There are 80,000 physicians, and if each took but one journal it would give to the 131 journals a circulation of 610 copies each, but the truth is that many take a number of journals. Only 75 of the journals are credited with a circulation of 1000 and over, and but 7 with a circulation of 5000 or more.

Dr. Louis Ottofy, of Chicago, has prepared the most complete and elaborate examination chart for dentists that we have seen. It is eleven by eighteen inches in size, with proper blanks for all parental and personal characteristics of the patient, and the fullest statistics of the condition, history, development and abnormalities of the teeth, to be indicated by symbols, and the whole so grouped as to indicate all desirable information at a glance. Comparison of sets of these, properly kept by dentists in different portions of the country, would prove of great interest and value to practitioners. It is probable that copies can be obtained of the author as above.

---

"But, friends, your manifestation of sympathy has aroused an echo across the sea. In view of the spontaneous and generous action of American brethren, I am speechless! It is utterly unmerited on my part! It can only be construed as a graceful testimony of fraternal good will towards the whole profession on this side the water. As such I accept it; I could even rejoice in my affliction if it should be the means of strengthening in any degree the bonds of mutual esteem and good understanding between English and American dentists."—*Reply of W. H. Waite to presentation address.*

---

Dr. T. B. Wheeler, of Chicago, who went abroad a few months ago in the expectation of locating for the practice of his profession in Europe, has returned, and desires henceforth to be known as an American dentist, practicing American dentistry in America. A careful survey of the field has convinced him that there is no place like home, and that Chicago is quite as much within reach of all the good things of the world as any spot upon its surface.

---

Professor John F. Weir, the well-known artist and critic, and head of the Yale Art School, will contribute to the August number of *Scribner's Magazine* a paper on the "Revival of Handicraft," which is a plea for the spread of skilled labor. The interest which the whole subject of manual training is exciting adds special timeliness to this article.

---

Dr. Geo. A. Maxfield has reduced the science of making hard rubber and corundum or emery disks to a very fine point. At the Montreal meeting he made them by the quantity, and they were of a fine quality too. This journal will give an exposition of his method, which is a very simple one, at an early day.

---

Dr. Joseph Lathrop, of Detroit, after occupying an office at 152 Woodward Ave. for more than a quarter of a century, has removed to No. 271 in the same street. Even after so long a time—twenty-six years—he could not make up his mind to leave the neighborhood.

---

Dr. F. B. Darby, of Elmira, sailed for Europe early in the month of July, for a summer's vacation. We most heartily commend him to the favorable attention of our *confreres*, on both professional and social considerations.

